# Discovery and Genomic Characterization of Noroviruses from a Gastroenteritis Outbreak in Domestic Cats in the US

**DOI:** 10.1371/journal.pone.0032739

**Published:** 2012-02-28

**Authors:** Pierfrancesco Pinto, Qiuhong Wang, Ning Chen, Edward J. Dubovi, Joshua B. Daniels, Laurie M. Millward, Canio Buonavoglia, Vito Martella, Linda J. Saif

**Affiliations:** 1 Department of Veterinary Public Health, University of Bari Aldo Moro, Valenzano, Italy; 2 Department of Veterinary Preventive Medicine, The Ohio State University, Wooster, Ohio, United States of America; 3 Department of Population Medicine, Cornell University, Ithaca, New York, United States of America; 4 Department of Veterinary Clinical Sciences, The Ohio State University, Columbus, Ohio, United States of America; Kantonal Hospital St. Gallen, Switzerland

## Abstract

Norovirus (NoV) RNA was detected in the stools of 6 out 14 (42.8%) 8–12-week-old cats with enteritis from a feline shelter, in New York State. Upon sequence analysis of the complete capsid, the six NoVs were found to be identical, suggesting the spread of a unique NoV strain in the shelter. The full-length genomic sequence (7839 nt) of one feline NoV, CU081210E/2010/US, was determined. In the capsid protein VP1 region, the virus displayed the highest amino acid identity to animal genogroup IV genotype 2 (GIV.2) NoVs: lion/Pistoia-387/06/IT (97.9%) and dog/Bari-170/07/IT (90.4%). These findings document the discovery of a novel feline calicivirus, different from vesiviruses, and extend the spectrum of NoV host range. Epidemiological studies using feline NoV-specific diagnostic tools and experimental infection of cats are required to understand whether NoVs have a pathogenic role in this species.

## Introduction

Noroviruses (NoVs) are non-enveloped, polyadenylated, single-stranded, positive-sense RNA viruses and represent a distinct genus in the family *Caliciviridae* [1]. Human NoVs have a worldwide distribution and represent the major cause of non bacterial gastroenteritis. The genome of NoV possesses three open reading frames (ORFs) encoding for a large non-structural polyprotein, a major capsid protein (VP1) and a small basic structural protein (VP2). Based on the full-length VP1, NoVs have been classified into five distinct genogroups (GI to V) [2]. GI, GII and GIV NoVs infect humans, with GII NoVs accounting for the majority of human infections [3]. NoVs have also been detected in animals and classified as GII (swine), GIII (ruminants), GIV (lions and dogs) and GV (mice) [1], [4].

Several studies have documented that NoVs also circulate in carnivores. NoVs were first identified in the feces of a captive lion cub with severe hemorrhagic enteritis in Italy [5]. Subsequently NoVs genetically related to the lion NoV were identified in the fecal samples of dogs with diarrhea in Italy and Greece [4], [6]. Based on the full-length VP1, the lion and dog NoVs were classified as a distinct genotype (GIV.2) within GIV, along with human Alphatron-like NoVs (GIV.1) [2]. Some canine NoVs are not classifiable within any established genogroups (GI-V), as they are distantly related to all other human and animal NoVs in the VP1 region [7]–[9].

Various viral agents including coronavirus, parvovirus, astrovirus, adenovirus, rotavirus and vesiviruses (feline calicivirus, FCV) have been identified in the stools of cats, although there is limited information on their role as enteric pathogens [10]–[13]. Small round structured viruses (SRSVs) have been detected occasionally in the feces of cats by using electron microscopy (EM) and have been mostly characterized as FCV [12], [14]. Interestingly, Norwalk-like 27-nm virus particles, clearly distinguishable from FCVs, were detected from an outbreak of vomiting and diarrhea in adult cats in Germany in 1987, although the viruses were neither adapted to cultivation *in vitro* nor characterized further [15]. More recently, antibodies specific for the baculovirus-expressed VP1 of Lion/NoV/Pistoia-387/06/IT were detected in the sera of cats by ELISA. These previous findings suggest that NoVs infect cats [16].

In this study, the stools of cats with diarrhea were screened for norovirus by reverse transcription (RT)-PCR with several calicivirus universal primer sets followed by sequence confirmation. Also, the full-length sequence of a feline NoV was determined for the first time.

## Materials and Methods

### Collection of samples

A total of 24 fecal samples were collected in 2010 from domestic cats (*Felis catus*) by the veterinary clinics of The Ohio State University (OSU) (10 samples, collection A) and Cornell University (CU) (14 samples, collection B). Diarrheic specimens were collected from hospitalized cats with gastroenteric symptoms. The 14 CU fecal samples, collected in July and August 2010, originated from young cats (8–12 weeks old) housed in a New York State animal shelter. The OSU cat specimens were collected in three different periods (July, November and December 2010) from animals of different ages (4 months to 8 years old) (Table 1). During the period of hospitalization, cats were located in single cages and feces were collected directly from the cat litter and identified individually with an alpha-numeric code. Fecal samples were stored at −20°C until processing. The fecal samples, collected during this study, were negative for common feline parasites.

**Table 1:** Feline sample collection and results for caliciviruses detection.

	SAMPLE CODE	ANIMAL ID	COLLECTION DATE	AGE^1^	SAMPLE TYPE	RT-PCR (p290-289)	RT-PCR (FNoV-F9 FNoV-R15)
**OSU (COLLECTION A)**	020911H	H	December 2010	4 M	FECES	-	**-**
	020911I	I	December 2010	1Y	FECES	-	-
	020911K	K	December 2010	2Y	FECES	-	-
	020911P	P	December 2010	1Y	FECES	-	-
	121310A	A	November 2010	2Y	FECES	-	-
	121310F	F	November 2010	8Y	FECES	-	-
	121310G	G	November 2010	3Y	FECES	-	-
	071510D	D	July 2010	8Y	FECES	-	-
	071510F	F	July 2010	3Y 3 M	FECES	-	-
	071510I	I	July 2010	1Y 4 M	FECES	-	-
**CU (COLLECTION B)**	081210A	A	July 2010	8W	FECES	-	NoV
	081210C	C	July 2010	8W	FECES	-	-
	081210D	D	July 2010	12W	FECES	FCV	-
	081210E	E	July 2010	8W	FECES	NoV	NoV
	081210F	F	July 2010	8W	FECES	FCV	-
	081210G	G	July 2010	8W	FECES	FCV	-
	081210H	H	July 2010	8W	FECES	FCV	-
	081210I	I	July 2010	8W	FECES	-	-
	081210J	J	July 2010	8W	FECES	NoV	NoV
	081210K	K	August 2010	8W	FECES	-	NoV
	081210L	L	August 2010	8W	FECES	-	-
	081210M	M	August 2010	8W	FECES	NoV	NoV
	081210N	N	August 2010	8W	FECES	-	-
	081210O	O	August 2010	8W	FECES	FCV	NoV

1 Y: year; M: month; W: week.

### RNA extraction

A 10% fecal suspension in 0.01 M phosphate buffered saline (PBS, pH 7.2) was made and the debris were removed by centrifugation at 16000× g for 3 min. The stool suspension was treated with DNase (RNase-Free DNase Set, Qiagen, Inc., Valencia, CA) and extracted using the RNeasy Mini Kit (Qiagen, Inc., Valencia, CA) and stored at −70°C.

### RT-PCR screening for calicivirus

The samples were analyzed by using broadly reactive primers p289–p290 targeting the highly conserved motifs (DYSKWDST and YGDD) of the RNA-dependent RNA polymerase (RdRp) [17]. Primer pair p289–p290 amplifies a fragment of 319 bp for NoVs and of 331 bp for vesiviruses and sapoviruses. Amplicons of the expected size (319 bp) were obtained from three samples (CU081210E, CU081210J and CU081210M). The amplicons were purified after gel excision with the QIAquick PCR Purification Kit (Qiagen, Inc., Valencia, CA) and sequenced directly from both directions. Upon sequence analysis of the 275 nt fragment, the three samples displayed 94% nt identity to the lion NoV/GIV.2/Pistoia-387/06/IT, confirming the diagnosis of NoV infection.

### Full-length genome sequencing of the feline NoV strain CU081210E

The primers are listed in Table 2. The 3′ end of the genome (∼3500 bp) was amplified with a 3′-RACE protocol [18] using specific forward primers (FNoV-F1 and FNoV-F2) designed based on the sequence of the p290/289 amplicons and reverse primers QO and QI. The internal fragment (∼2000 bp) was amplified using specific reverse primers (FNoV-R1 and FNoV-R2) and the forward primer P1210, targeting the NTPase region [19]. The 5′end fragment was obtained using specific reverse primers (FNoV-R5 and FNoV-R6) and the primer FragAF [20]. The actual 5′-end of the genome was generated with the 5′-RACE System for Rapid Amplification of cDNA Ends, Version 2.0 (Invitrogen, Corp., Carlsbad, CA) using a 5′-RACE protocol with minor modifications [21] and specific reverse primers (FNoV-R10 and FNoV-R11). The cDNA was synthesized using SuperScript III First-Strand cDNA Synthesis Kit (Invitrogen, Corp., Carlsbad, CA) and PCR was performed using TaKaRa Ex Taq™ polymerase (TaKaRaMirus Bio, Madison, WI).

**Table 2:** Oligonucleotides used for cDNA synthesis and amplification in this study.

Oligonucleotide	Polarity	Position^a^	Sequence (5′ to 3′)^b^	Note^c^	Reference
FNoV-F1	+	4480–4503	GGTTATTAAGGCCGCGCTTGACAT	A	This study
FNoV-R1	−	4659–4681	GGCTGAGAGGGTGTAAATCCAGT	A	This study
FNoV-F2	+	4507–4530	GGTTAAATTCTCTGCCGAACCCGA	A	This study
FNoV-R2	−	4626–4648	GTTGACTTGAGAGGTGCAGGGAA	A	This study
P1210	+	1610–1632	GGICMICCIGGIWKIGGIAARAC	A	[19]
FNoV-F3	+	5143–5163	CGTGCCCAAGTTCGAAGCCAT	S	This study
FNoV-R3	−	7251–7274	CATTCCAGTCAACTAGCGTGGTCA	S	This study
FNoV-F4	+	3434–3455	GTCAAACGTGCCAGTGGTGAAC	S	This study
FNoV-R4	−	2826–2846	CACTGAGTCCTTTACTGGAGA	S	This study
FNoV-R5	−	2797–2815	CTTTCGCCCACGACCCTTC	A	This study
FNoV-R6	−	2787–2805	CGACCCTTCTTGCTCTTAC	A	This study
FNoV-R7	−	4197–4216	CTTAAGGGCTGCGGTGAATC	S	This study
FNoV-R8	−	6525–6546	GCAGATTATACTCCTGGTACTG	S	This study
FNoV-F5	+	5995–6014	ATGAGGCCCAGGTTGTGCAC	S	This study
FragAF	+	1–21	atattaattaaGTGAATGAAGATGGCGTCTAA	A	[20]
FNoV-F6	+	680–699	GCACTCTACAAACTCAATGG	S	This study
FNoV-R9	−	532–554	CGAGGTAGATGGCGTAGTGGTAG	S	This study
FNoV-R10	−	498–518	ACATCTCGAGGATGGAGCCAG	A	This study
FNoV-R11	−	424–446	GTTTGACTTCACGCTCAGACAGG	A	This study
FNoV-R12	−	2099–2115	GTGTTGCCAGCCTTGTC	S	This study
FNoV-F9d	+	4655–4677	GCCCACTGGATWTACACCCTCTC	A	This study
FNoV-R14d	−	7067–7088	CYT GGT TRT ACC CAA ACT CCA C	A	This study
FNoV-R15	−	4970–4993	CTG ATG GTT GGG TCC TCT GGT CCA	A	This study
P290d	+	4445–4467	GATTACTCCASSTGGGAYTCMAC	A	[17]
P289d	−	4742–4763	TGACGATTTCATCATCMCCRTA	A	[17]
Q_T_	+/−	3′/5′end	CCAGTGAGCAGAGTGACGAGGACT CGAGCTCAAGCTTTTTTTTTTTTTTTTT	A	[18]
Qo	+/−	3′/5′end	CCAGTGAGCAGAGTGACG	A	[18]
Q_I_	−	3′end	GAGGACTCGAGCTCAAGC	A	[18]
AAP	+	5′end	GGCCACGCGTCGACTAGTACGGGI IGGGIIGGGIIG	A	[21]
AUAP	+	5′end	GGCCACGCGTCGACTAGTAC	A	[21]

a Nucleotide position refers to the complete genome sequence of the feline NoV CU081210E (GenBank accession no. JF781268).

b Non viral sequences are indicated in lowercase.

c A: amplification; S:sequencing.

### Cloning and sequencing

The RT-PCR or PCR products were purified using the gel purification kit (Qiagen, Inc., Valencia, CA) and cloned into pCR-XL-TOPO vector (Invitrogen, Corp., Carlsbad, CA) for sequencing. DNA sequencing was performed using BigDye Terminator Cycle chemistry and an automated sequencer ABI Prism 3100XL (Applied Biosystems, Foster, CA).

### Sequence analysis

Sequence editing, assembling and alignment were performed using BioEdit Sequence Analysis Editor (version 7.0.9.0) [22]. Basic Local Alignment Search Tool (BLAST, http://www.ncbi.nlm.nih.gov) was used to find homologous hits in the sequence databases. Phylogenetic analysis (Neighbor-Joining) with bootstrap (1,000 replicates) was conducted using MEGA version 5.03 [23]. Identity matrices were calculated without removing the gaps and with no distance correction.

Pair-wise identity in the full-length VP1 of strain CU081210E to 180 NoV strains was determined using multiple alignments generated with Bioedit software package vers. 2.1 [22]. The values were calculated by the uncorrected distance method using a 181-sequence alignment without removing the gaps, following the outlines of Zheng et al. [2].

Pair-wise identity in the partial polyprotein (∼250 aa) was also calculated using a selection of 72 reference NoV strains for whom either partial (the C-terminus of the polyprotein) or complete ORF1 sequences are available. In addition, the full-length polyprotein was aligned to cognate sequences of five reference NoV strains and the cleavage sites were predicted on the basis of conserved aa motives [24], [25].

The full length genome sequence of the feline GIV NoV CU081210E/US was deposited in GenBank with accession number JF781268.

### RT-PCR for the diagnosis of feline NoVs

An RT-PCR specific for feline NoV was developed based on the cat and lion NoV sequences. Primers FNoV-F9 and FNoV-R15 (Table 2) were designed to amplify a 338-bp amplicon at the 3′end RdRp region. The assay was performed using the QIAGEN one-step RT-PCR kit (Qiagen, Inc., Valencia, CA). The amplification program included 50°C for 30 min, 95°C for 15 min, 35 cycles at 94°C for 30 sec, 57°C for 30 sec and 72°C for 60 sec and a final extension of 10 min at 72°C.

### RT-PCR amplification of the ORF2 of feline NoVs

Primers FNoV-F9 and FNoV-R14 were designed to amplify a 2433 bp fragment encompassing the 3′ end of ORF1, the full-length ORF2 and the 5′ end of ORF3. The amplification was performed using the QIAGEN one-step RT-PCR kit (Qiagen, Inc., Valencia, CA). The thermal conditions consisted in 50°C for 30 min, 95°C for 15 min, 35 cycles at 94°C for 30 sec, 53°C for 30 sec and 72°C for 2 min and 30 sec and a final extension of 10 min at 72°C. The primers are listed in Table 2.

## Results

### Full-length genome sequencing of the feline NoV strain CU081210E

**Figure 1:**
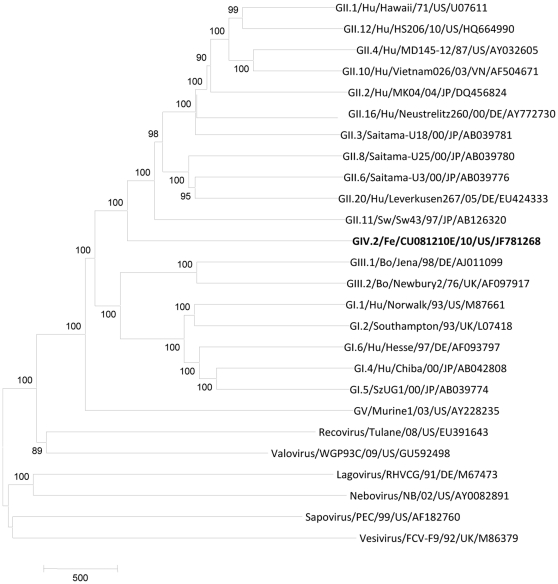
Neighbor-Joining phylogenetic tree of caliciviruses based on the complete genomes (nucleotide). The newly identified feline NoV CU081210E/10/USA is in bold. Bootstrap values are shown near branches.

In RT-PCR with primers p289/p290, 8 out of the 24 feline samples (all originated from CU collection B), were positive, but only 3 samples (CU081210E, CU081210J and CU081210M) showed a band of the expected size for NoV (319 bp), while the other 5 samples showed a band of 331 bp and were confirmed as FCV by sequence analysis (Table 1). Upon sequence analysis, the feline NoV strains were highly similar to the lion GIV NoV Pistoia-387/06/IT (94% nt and 98% aa identity) in the 275 nt fragment. By the 3′ RACE protocol, a ∼3.5 kb fragment (from the 3′end of ORF1 through the poly-A tail) was obtained only for strain CU081210E. The 7839-bp complete genome sequence of the cat NoV was determined by a primer walking strategy. By comparison with a selection of full-length genomic sequences of GI, GII, GIII and GV NoV strains available in the databases, the highest nt identity was found to GII NoVs (56.4–57.6% nt). Nucleotide identity to GI, GIII and GV NoV was ≥47.1%, while identity to other caliciviruses (sapovirus, vesivirus, lagovirus, nebovirus, recovirus and valovirus) ranged from 29.3 to 34.5% (Table 3, Figure 1).

**Table 3:** Noroviruses identity (%) to the new feline strain fe/CU081210E/10/USA.

		GI	GII	GIII	GIV.1	GIV.2 lion (EF450827)	GIV.2 dog (EU224456)	GV	GVI	
**feline/NoV/GIV.2/CU081210E/US/2010 (JF781268)**	**ORF1** *	60,8–64,4	62,2–69,5	59,8–64,2	69,2–70,3	94,5	85,7	55,7	79,0–86,3	Nucleotide sequence
		62,5–65,1	67,3–75,3	62,5–65,1	76,0–78,0	98,9	98,4	51,2	95,3–98,4	Amino acid sequence
	**ORF2**	43,3–46,2	50,2–53,9	41,4–43,9	64,6–65,2	94,1	82	46,2	52,8–59,7	Nucleotide sequence
		37,6–41,5	45,5–49,8	36,7–37,4	68,2–68,5	97,9	90,4	36,9	50,2–54,5	Amino acid sequence
	**ORF3**	26,6–28,2	39,0–47,8	31,9–32,9	58,4–59,3	93,9	72,9	25,2	46,3–47,2	Nucleotide sequence
		25,4–30,3	34,7–44,3	20,9–26,1	58,8–60,7	96,8	77,6	20,2	41,5–44,7	Amino acid sequence

* The ORF1 identity (%) was calculated on the partial RdRp nucleotide (∼750 nt) and amino acid (∼250 aa) sequence of 72 representative strains available in GenBank.

Three ORFs were identified in the genome of strain CU081210E, as described for other NoVs [1]. The ORF1 was 5241 nt long and encodes the non-structural polyprotein with a predicted size of 1747 aa. In the polyprotein, 5 cleavage sites were predicted: Gln^367^/Gly^368^, Glu^735^/Gly^736^, Glu^922^/Gly^923^, Glu^1055^/Ala^1056^ and Glu^1236^/Gly^1237^ located between the N-terminal protein, NTPase, “3A-like” protein, VPg, protease and RdRp, respectively [24], [25].

As most diagnostic primers target highly conserved motives in the RdRp (ORF1), partial sequences of the 3′ end of the RdRp are available in the databases. A selection of 72 partial sequences of the RdRp (∼250 aa) of NoVs was retrieved from GenBank and used to compare in detail the feline NoV strain. Identity was 98.9% aa and 94.5% nt to the strain lion/Pistoia-387/06/IT and 98.4% aa and 85.7% nt to strain dog/Bari-170/07/IT. Identity to human GIV NoVs was 76–78% aa and 69.2–70.3% nt (Table 3).

**Figure 2:**
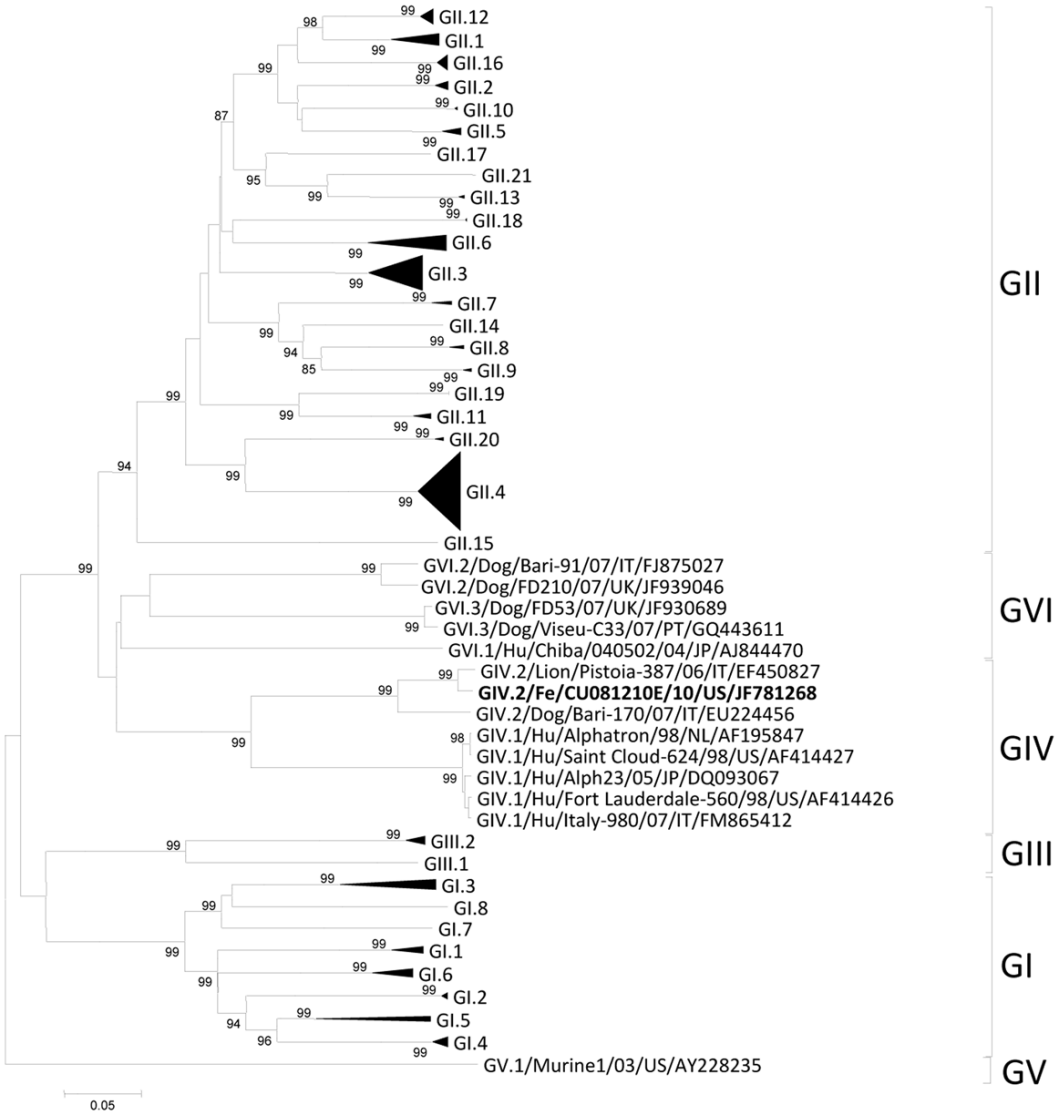
Neighbor-Joining phylogenetic tree of noroviruses based on the complete capsid sequence (amino acid). The newly identified feline NoV CU081210E/10/US is in bold. Bootstrap values are shown near branches.

The ORF2 was 1737 nt long and the predicted size of VP1 was 579 aa. The identity was calculated on a selection of 181 NoV capsid sequences. The feline NoV displayed 97.9% aa and 94.1% nt identity to the strain lion/Pistoia-387/06/IT, 90.4% aa and 82% nt to the NoV dog/Bari-170/07/IT and 64.6–68.5% to human GIV (Alphatron-like) NoVs. Identity to non-GIV NoVs was 36.7–54.5% aa (Table 3, Figure 2). In the VP1, four regions were identified, namely the NH2-terminal arm (residues 10–45), the S-domain (residues 46–221), the P1-subdomain (residues 222–274 and 451–569) and the P2-domain (residues 275–450) [26]. In the highly variable P2-domain, identity to the lion and to the dog strains (Bari-170/07/IT) was 97.1% and 86.3% aa, respectively, while identity to human GIV NoVs was much lower (∼44% aa). A 20-aa insertion was present in the P2-domain of animal GIV NoVs, with respect to human GIV NoVs.

The ORF3 and the predicted minor structural protein (VP2) were 765 nt and 255 aa long. In the ORF3, identity was high to the lion NoV (96.8% aa and 93.9% nt) and the canine strain Bari-170/07/IT (77.6% aa and 72.9% nt), while it was lower to the other NoVs strains (Table 3).

A 17-nt overlap was present between the end of ORF1 and the beginning of ORF2, while there was a 1-nt overlap between ORF2 and ORF3. The 5′ untranslated region (UTR) was 10 nt-long, while the 3′ UTR was104 nt long.

### RT-PCR for the detection of feline NoVs

Using the sequences of the feline NoVs obtained in the study, a new diagnostic RT-PCR was developed. By re-screening the sample collections A and B with primers FNoV-F9 and FNoV-R15, 3 additional samples tested positive to NoV from the collection B, but none from collection A. Accordingly, 6 out of the 14 feline diarrheic samples (42.8%) from Cornell University veterinary clinic were positive for NoV. These samples were obtained from 8–12 weeks old kittens with enteric signs housed in a New York shelter (Table 1).

The sequences of a 2.4 kb-genomic fragment of three additional feline NoV strains (CU081210J, CU081210M, CU081210O) were obtained and compared with the sequence of strain CU081210E. The four feline NoV strains displayed 100% nt identity to each other, suggesting a clonal origin.

## Discussion

Feline vesiviruses, commonly referred to as FCV, are widespread in cats and they are associated with mild to severe disease of the upper respiratory tract [27]. FCV may be detected from conjunctival, nasal and oropharyngeal swabs and from internal organs [12], [28]. Although it is not clear whether FCV also plays a role as an enteric pathogen or if it is shed in the feces after primary localization in other regions, it has been occasionally identified in the feces of cats and associated with enteric disease [28]–[31]. Also, differences in resistance to bile acids have been found between FCV strains of enteric and respiratory origin, thus suggesting that enteric FCVs have the ability to replicate actively in the enteric tract while respiratory FCVs do not [14]. By EM observation, SRSVs have been detected in 6% of the stools of both symptomatic and asymptomatic cats [12]. In the present study FCVs were identified by RT-PCR in 5 out of 14 (35.7%) diarrheic fecal samples of group B while they were not detected from animals of group A (Table 1).

Also, in this study, a novel calicivirus was discovered in cats and characterized as a member of the Norovirus genus by sequence analysis of the complete genome. This novel feline calicivirus was detected in 6/14 samples collected from cats housed in a shelter in New York State (collection B) (Table 1). These findings are in agreement with previous evidence that cats can be infected with NoVs [15], [16]. It is of relevance that animals from group B showed enteric symptoms and were young kittens almost of the same age (8 to 12 weeks old). This age period is critical for kittens, since maternally derived antibodies tend to wane and the animals become fully susceptible to various pathogens [32]. On the other hand, NoVs were not detected in animals from group A. This might reflect either geographic or temporal variations, age-related patterns of susceptibility or merely the relatively small number of samples included in the analysis.

The complete sequence of one strain (CU081210E) and partial (∼2.4 kb) sequences of three additional strains (CU081210J, CU081210M, CU081210O) were analyzed. All the NoV strains displayed 100% nt identity to each other. The clonal origin of these viruses suggests that a single NoV strain was spreading quickly in group B animals, consistent with the highly infectious nature of NoVs [33].

In the VP1, the feline NoV displayed high aa identity to the GIV.2 NoV strain lion/Pistoia-387/06/IT (97.9%) and to the dog strain Bari-170/07/IT (90.4%) while the aa identity was ∼68% to human GIV.1 (Alphatron-like) NoVs (Table 3). Based on the currently accepted classification system (*2*), the feline NoV can be classified as a GIV.2 genotype (Figure 2). In the hypervariable P2-domain, the feline NoV strain was highly conserved with respect to the NoV strains lion/Pistoia-387/06/IT and dog/Bari-170/07/IT (97.1% and 86.3% aa, respectively) while it was less related to human GIV NoVs (∼44% aa). The P2-domain represents the protruding region of the capsid protein and is the binding site for cells and protective antibodies [34], [35].

A fragment of the RdRp (the 3′end of the ORF1) was used for analysis and comparison with a large selection of NoV strains belonging to all genogroups. This part of the NoV genome can be sequenced easily using the 3′-RACE protocol and forward primers designed in conserved aa motives of the RdRp. Therefore, sequence data spanning this region is available for most NoV strains. In the RdRp the feline NoV displayed high identity to animal GIV.2 NoV strains and to other unusual canine NoV strains that are highly divergent in the VP1 and likely constitute a novel NoV genogroup (GVI) [7], [9].

Although a number of GIV NoVs have been detected in humans and sewage samples thus far [36], [37], the full-length genome sequence of GIV NoVs is not available for detailed comparison with other NoV genogroups. In this study the complete genome sequence of a GIV NoV was determined and compared with full-length genomic sequences of NoV strains available in the databases, revealing the highest nt identity to GII NoVs (56.4–57.6% nt), whilst nt identity to GI, GIII and GV NoV was ≤47.1% (Table 3, Figure 1). This confirms that NoV genogroups GII and GIV are genetically much more related to each other than to other NoV genogroups. The GIV includes both animal (carnivores) GIV.2 and human GIV.1 viruses [6], [7]. Human GIV.1 NoVs are identified only sporadically although sewage-based surveillance studies conducted in some countries suggest that these NoVs are rather common [37]. Human GIV.1 NoVs are more related to carnivore GIV.2 NoVs than to other humans NoV genogroups (GI and GII), thus suggesting an intersection during the evolution of GIV NoVs, likely due to the social interactions of humans with carnivores during the process of domestication and to the possibility of heterologous infections, as demonstrated under experimental conditions [38], [39].

In conclusion, the results of this study demonstrate that: i) members of two distinct calicivirus genera (vesivirus and norovirus) infect domestic cats; ii) NoVs detected in cats are similar to NoVs found in other carnivores (lions and dogs), thus suggesting inter-species circulation for GIV.2 NoV in these animal species. Larger epidemiological investigations and animal experiments are warranted to assess firmly the role of NoV infections in cats. Also, these findings pose a challenge for epidemiological studies of feline enteric pathogens, highlighting the need for reliable NoV-specific diagnostic assays.
